# Australians’ views and experience of personal genomic testing: survey findings from the Genioz study

**DOI:** 10.1038/s41431-018-0325-x

**Published:** 2019-01-21

**Authors:** Jacqueline Savard, Chriselle Hickerton, Rigan Tytherleigh, Bronwyn Terrill, Erin Turbitt, Ainsley J. Newson, Brenda Wilson, Kathleen Gray, Clara Gaff, Anna Middleton, Elaine Stackpoole, Sylvia A. Metcalfe

**Affiliations:** 1 Deakin University, School of Medicine, Faculty of Health, Geelong, Victoria Australia; 2 The University of Sydney, Faculty of Medicine and Health, Sydney School of Public Health, Sydney Health Ethics, Sydney, NSW 2006 Australia; 3 Garvan Institute of Medical Research, Sydney, Australia; 4 Genetics Education and Health Research, Murdoch Children’s Research Institute, Melbourne, Australia; 5 Department of Paediatrics, The University of Melbourne, Melbourne, Australia; 6 Genome.One, Sydney, Australia; 7 St. Vincent’s Clinical School, University NSW, Sydney, Australia; 8 School of Epidemiology and Public Health, University of Ottawa, Ottawa, Canada; 9 Health and Biomedical Informatics Centre, The University of Melbourne, Melbourne, Australia; 10 The Walter and Eliza Hall Institute of Medical Research, Melbourne, Australia; 11 Society and Ethics Research, Connecting Science, Wellcome Genome Campus, Cambridge, UK; 12 Present Address: National Human Genome Research Institute, Bethesda, MD USA; 13 Memorial Hospital, St John’s, Newfoundland, Canada; 14 Genetic Services of Western Australia, Subiaco, WA Australia

**Keywords:** Ethics, Personalized medicine

## Abstract

Personal genomic tests (PGTs) for multiple purposes are marketed to ostensibly healthy people in Australia. These tests are generally marketed and purchased online commercially or can be ordered through a health professional. There has been minimal engagement with Australians about their interest in and experience with ordering a PGT. As part of a multistage, interdisciplinary project, an online survey (Stage 2 of the Genioz study) was available from May 2016 to May 2017. In total, 3253 respondents attempted the survey, with 2395 completed Australian responses from people with and without experience of having a PGT: 72% were female; 59% of the whole sample were undertaking/or had a university education; and, overall, age ranged from 18—over 80. A total of 571 respondents reported having had a genetic test, 373 of these classifiable as a PGT. A bivariate analysis suggests people who have undergone PGT in our sample were: women aged 25 and over; or in a high socioeconomic group, or have a personal or family diagnosis of a genetic condition (*P* ≤ 0.03). After a multivariate analysis, socioeconomic status and a genetic condition in the family were not of significance. The most common types of PGT reported were for carrier status and ancestry. Findings suggest greater awareness of, and an increasing demand for non-health related PGT in Australia. To support both consumers and health care professionals with understanding PGT results, there is a need for appropriate support and resources.

## Introduction

It is now possible to obtain personal genomic information outside the context of a clinical or hospital setting, for example, through purchasing a personal genomic test (PGT). These tests can be grouped into health-related and recreational-associated tests. Health-related PGTs include those that test for a predisposition to certain conditions, responses to drugs, carrier status, and wellness (encompassing holistic aspects, such as diet and nutrition, intolerances, and allergy susceptibility) [1]. So called recreational PGTs examine family relationships (including ancestry, ethnicity, and paternity), fitness and sporting abilities, physical and personality/behavioral traits, and testing for dating compatibility [2].

Markets for the different types of PGT are variable, depending on the geographical location and the regulatory environment in which the company and consumers are located. In Australia, the market for PGT has emerged at a slower pace compared to other countries [3]. Currently, Australian consumers can access PGT with or without a health care professional depending on the type of test they are seeking. They can obtain a test online from both onshore and overseas providers.

There are now several public attitude studies that explore the online personal genomics market [4, 5]. However, findings from these studies reflect the regions in which they were undertaken and are also a product of the social, political, and health care systems of the countries in which the research was conducted [4, 6, 7]. Research to date examining consumer knowledge, attitudes, and experiences of PGT in Australia has been limited. Findings from early research reported a small interest in direct-to-consumer PGT [8] with greater trust in receiving genomic information through a health care professional compared with a commercial company [9]. Accessibility of genomic tests has increased worldwide [10] and the presence of genomics on the national health policy agenda for Australia has also increased with a $500 million commitment over the next ten years to an Australian Genomics Health Futures Mission [11]. Therefore, while earlier studies offered some insights into Australians’ views, a wider examination of PGT in Australia from prospective and current consumers is due.

We sought to examine this area through the Genioz study, which was initiated in 2015. An outline of the Genioz study (www.genioz.net.au), is described elsewhere [12] but briefly this included five stages of data collection and analysis: focus groups, online survey, interviews, deliberative workshops, and ethical critique. In this paper, we report on the findings from the year-long nationwide survey (stage 2) quantitatively exploring Australians’ awareness, attitudes and experiences of PGT.

## Materials and methods

The survey was approved by The University of Melbourne Human Research Ethics Committee (ID 1545806.1). At the end of the survey, participants could nominate whether they would like to participate in an interview about their views or experiences regarding personal genomic testing. This paper reports the survey findings only and interview data will be reported separately.

### Survey design

A cross-sectional online survey was developed in REDCap comprising seven sections (see SF 1). Survey sections contained a mix of question and answer formats including: radio button (single answer), Likert scales, check-box/multiple response, and open-text boxes. Branching logic throughout the survey was based on respondents’ experiences of testing. Therefore, respondents may not have answered all the same questions and the time taken to complete the survey would have varied.

Survey questions were informed by the Genioz focus groups (stage 1), team expertize, and relevant published literature [4, 13–18]. A large portion of the survey focused on respondent experiences with PGT. However, the language used in the questions relating to experience with testing was framed as “genetic testing”. The research team felt this was a more familiar phrase for respondents to understand. Questions collected subjective attitude and opinion data, which are difficult to validate using traditional methods. A modified Delphi technique was used for face validity and consisted of two rounds to refine question inclusion [19]. Round 1 involved face-validity testing via a panel of 15 experts (including some members of the research team) who were asked to independently review each survey item based on the extent to which they were relevant to the aims of each section, and how difficult each item may be for a nonexpert to answer. Experts were from the following disciplines: health communication research, genetic counseling, law, public health and genetic research, genetics education, science communication, program evaluation, clinical genetics, and bioethics.

The panel’s round 1 Delphi responses were reviewed by the research team followed by modification of the survey items. Round 2 consisted of further face-validity testing via ten of the experts, followed by additional consultation within the research team leading to final item alteration. Research team members, as well as other colleagues, answered the survey in demonstration mode on different technological platforms to test for access and functionality. In addition, the survey content was piloted with participants from the initial Genioz focus groups [12] (who are members of the public and potential consumers of PGT). Key definitions were provided throughout the survey, for example: a definition of PGT, pharmacogenetics, and carrier testing.

### Survey recruitment

A study website was developed to host information such as: study aims, frequently asked questions related to survey participation; a link to the Participant Information Statement and the embedded study survey. Once read, a respondent wishing to consent to participating (and having confirmed they were ≥18 years) could enter the survey. The survey was only available to those who had access to the Internet (accessible on multiple device types). Once the survey was completed, respondents could follow a link to some balanced information about PGT if they wanted more information.

A communication strategy developed by research team members guided the production of various online and hard copy materials to advertise the survey to members of the public (SF 2). Firstly, communication teams of the partnering institutes and universities assisted in promoting the launch of the website and survey via their online platforms and newsletters. To broaden recruitment opportunities, hard copy materials advertising the survey were sent to various community groups such as genealogical societies, recreational, and sport-related groups. Additionally, the survey was promoted on the social media platforms Facebook and Twitter [19]. Thought provoking questions with hashtags (such as: *How much do you think a person’s #genes contribute to their personality and other attributes? What are your thoughts about genetic tests that can be bought online? Tell us in our #survey*) were posted and accompanied by images, cartoons or custom-made memes to pique the public’s interest and prompt survey participation. As such, recruitment of the survey used a mix of targeted, convenience and snowball sampling.

Our aim was to recruit a cross-section of Australian publics according to census data. At the 6-month recruitment point the research team noticed that some demographic characteristics were under represented. These included: a lower proportion of individuals from a lower education status; respondents from the 40–64-year-old age range, and males. Following approval of an ethics amendment and sign off on contract negotiations a market research company, Survey Sampling International (SSI), assisted recruitment from these groups. These data collection occurred at the 10-month recruitment point.

### Data analysis

Survey data were prepared for analysis by removing blank and duplicate records. Respondents were included if they were ≥18 years and resided in Australia according to the postcode stated, therefore excluding people who reported they lived overseas. Complete and partial surveys were included for analysis; thus, *n* varies by question. Descriptive and logistic regression analyses were performed in Stata 14.2 [20].

Open-ended responses were recategorized once consensus was reached within the research team. Responses were either added to existing categories or a new category was created. For example, recoding was necessary to categorize whether people had had a PGT or a clinical test if they reported having had carrier testing, which can be offered in both contexts in Australia. Therefore, carrier tests were NOT classified as a PGT when respondents had indicated the following: (1) they either had a diagnosed genetic condition themselves and/or within their family/had a family history; or (2) they had carrier testing through a research program. Carrier tests were then classified as PGT if: there was NO diagnosed genetic condition in themselves or their family; or when asked about why they had carrier testing, they indicated one or more of the following reasons: (a) “I was planning to have children and wanted to know if there is a risk of passing on a genetic condition to my children”; (b) “I wanted this information for my grandchildren”; (c) “I thought I was at a higher risk because of my ethnic background”; (d) “I was curious”; and (e) “I didn’t think about it, I just did it”.

This survey was exploratory and not hypothesis driven. A Pearson chi-squared test was carried out to explore the relationship between age and test type; bivariate logistic regression sought to investigate which demographic variables and characteristics may be associated with experience of testing. Analyses were performed using multivariate logistic regression to adjust for potential confounding sociodemographic variables found to be different between those who had PGT and those who had not.

## Results

### Respondent demographics

From May 2016 to May 2017, there were 3253 attempts to start the survey. Of these, 2841 were unique attempts by respondents living in Australia, with 2395 completed surveys. Respondents living overseas (*n* = 101 from 32 countries) were excluded from analysis. The demographic characteristics of respondents are described in Table 1. Of respondents who completed the demographic section, 72% were women. The age range was from 18 years to over 80 and 59% of the whole sample had either previously studied or were studying at university. By comparison with the Australian Bureau of Statistics 2011 census data (SF 3), our sample contains a greater proportion of respondents from higher socioeconomic indexes for areas (SEIFA) categories and captured a greater proportion of respondents from the age categories of 20–24 years and 55–65 years (SF 4).

**Table 1:** Demographic characteristics of respondents to the Genioz online survey

Demographic variable	All respondents	Yes, had genetic testing of any type	Not tested/unsure if had testing
	*n* (%)	*n* (%)	*n* (%)
*Age* ^a^	*n* = 2819^b^	*n* = 571^b^	*n* = 1709^b^
18–24 years	407 (14.4)	30 (5.3)	320 (16.2)
25–49 years	1161 (41.2)	241 (42.2)	808 (40.8)
50+ years	1251 (44.4)	300 (52.5)	851 (43.0)
*Gender*	*n* = 2819		
Male	786 (27.9)	122 (21.4)	610 (30.8)
Female	2018 (71.6)	447 (78.3)	1357 (68.6)
Other/I prefer not to say	15 (0.5)	2 (0.4)	12 (0.6)
*Highest level of education*	*n* = 2815		
Never studied at university	1144 (40.6)	181 (31.8)	863 (43.6)
Currently studying/completed university	1671 (59.4)	388 (68.2)	1115 (56.4)
*SEIFA (ISRAD)*	*n* = 2797		
1 (most disadvantaged)	266 (9.5)	54 (9.5)	196 (10.0)
2	295 (10.5)	64 (11.2)	207 (10.5)
3	496 (17.7)	79 (13.9)	371 (18.9)
4	638 (22.8)	113 (19.9)	461 (23.4)
5 (most advantaged)	1102 (39.4)	259 (45.5)	731 (37.2)
*Working in genomics and/or life sciences*	*n* = 2819		
Yes	420 (14.9)	118 (20.7)	267 (13.5)
No	2399 (85.1)	453 (79.3)	1712 (86.5)
*Parent*	*n* = 2819		
Yes/No, I’m currently pregnant	1653 (58.6)	411 (72.0)	1097 (55.4)
No	1161 (41.2)	160 (28.0)	877 (44.3)
I don’t know	5 (0.2)	0	5 (0.3)
*Adopted* ^b^	*n* = 2814		
Yes	61 (2.2)	13 (2.3)	40 (2.0)
No/I don’t know	2753 (97.8)	552 (97.7)	1926 (98.0)
*Spirituality* ^b^	*n* = 2785		
Yes	1057 (38.0)	200 (35.7)	761 (38.9)
No/I’m not sure	1725 (62.0)	360 (64.3)	1197 (61.1)
*Genetic condition diagnosed in self*	*n* = 2819		
Yes	384 (13.6)	162 (28.4)	175 (8.8)
No/I don’t know	2435 (86.4)	409 (71.6)	1804 (91.2)
*Genetic condition diagnosed in family*	*n* = 2819		
Yes	680 (24.1)	231 (40.5)	378 (19.1)
No/I don’t know	2139 (75.9)	340 (59.5)	1601 (80.9)
*Self-reported health (SF-36)*	*n* = 2819		
Excellent	364 (12.9)	87 (15.2)	250 (12.6)
Very good	1039 (36.9)	213 (37.3)	729 (36.8)
Good	925 (32.8)	162 (28.4)	659 (33.3)
Fair	366 (13.0)	75 (13.1)	262 (13.2)
Poor	122 (4.3)	33 (5.8)	77 (3.9)
Unknown	3 (0.1)	1 (0.2)	2 (0.1)
*Recruitment source*			
Main survey	2053 (72.8)	529 (92.6)	1282 (64.8)
SSI survey	766 (27.2)	42 (7.4)	697 (35.2)

^a^Age categories were collapsed to reflect age categories in stage 1 focus groups^12^

^b^Optional question to answer

Of the respondents who answered questions about their occupation, 420 (14.9%) confirmed they worked in life sciences and/or genomics. Overall, 384 (13.6%) respondents reported having a diagnosed genetic condition themselves and 680 (24.1%) reported knowing about a diagnosed genetic condition within their family. Knowledge questions were answered well overall, with a median of 12 out of 15 questions answered correctly (See SF 5, Boxplot of average median score).

### Respondents’ experience with testing

**Fig. 1:**
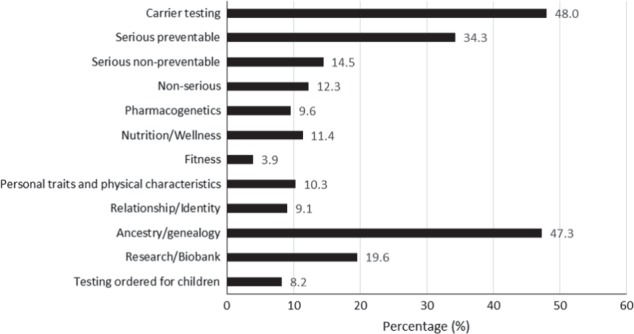
Types of tests reported by respondents (**a**, **b, c**) (*n* = 571). **a** The order of types of tests as they appeared in the survey. **b** Respondents could tick more than one test. **c** “Other” types of testing included: School

In this sample, 571 individuals (22.4%) reported having had a genetic test. A further 1979 (77.6%) had never had a genetic test or were unsure if they ever had testing. Of the 571 who had testing, 373 (65.3%) had a PGT as defined by the research team. Ancestry and carrier testing were the two most common types of testing reported by respondents who stated having any type of test (see Fig. 1). The most common way testing was arranged was by purchasing by self, online (SF 6). This may reflect the high volume of ancestry testing reported, which is usually purchased online.

Results of a bivariate logistic regression analysis exploring the relationship between demographics and respondents who had reported having a PGT are shown in Table 2 (this includes carrier testing as a PGT, *n* = 373). The demographic characteristics showing an association with having pursued PGT include: being female (*P* = 0.002); belonging to one of two distinct age groups, either 25–49 (*P* < 0.001) or over 50 (*P* < 0.001); living in a SEIFA in the highest quintile (*P* = 0.03) (indicating high socioeconomic advantage); currently studying at university or having been university educated (*P* < 0.001); working in the field of genomics and/or the life sciences (*P* ≤ 0.001); being a parent (including currently pregnant) (*P* < 0.001); having a diagnosed genetic condition in self (*P* < 0.001); or having a diagnosed genetic condition in the family (*P* < 0.001). A multivariate logistic regression was conducted to account for potentially confounding variables. From this analysis, the variables of SEIFA and having a diagnosed genetic condition in the family were no longer associated with having had a PGT (see Table 2 adjusted odds ratio).

**Table 2:** Bivariate logistic regression and multivariate logistic regression analysis of demographics and testing experience with carrier testing included as a PGT^a^

Variable	Had PGT *n* (%)	Not had PGT *n* (%)	Unadjusted odds ratio [95% CI]	*P* value	Adjusted odds ratio [95% CI]	*P* value
*Age* ^b^						
50+	222 (20.7)	851 (79.3)	*ref*.			
25–49	140 (14.8)	808 (85.2)	0.7 [0.5–0.8]	<0.001		
18–24	11 (3.3)	320 (96.7)	0.1 [0.07–0.5]	0.001		
*Age*						
18–49	151 (11.8)	1128 (88.2)	*ref*.			
50+	222 (20.7)	851 (79.3)	1.9 [1.2–2.0]	<0.001	2.2 [1.7–2.9]	<0.001
*Sex*						
Male	85 (12.2)	610 (87.8)	*ref*.			
Female	287 (17.5)	1357 (82.5)	1.5 [1.2–2.0]	0.002	1.6 [1.2–2.1]	0.001
*SEIFA (IRSAD)*						
First to fourth quintiles	212 (14.7)	1235 (85.3)				
Fifth quintile	161 (18.1)	730 (81.9)	1.3 [1.0–1.6]	0.03	1.2 [0.9–1.5]	0.1
*Education*						
Never studied at university	115 (11.9)	863 (88.2)	*ref*.			
Currently studying/completed university	256 (18.7)	1 115 (83.0)	1.7 [1.4–2.2]	<0.001	2.3 [1.8–3.1]	<0.001
*Working in genomics and/or life sciences*						
No	333 (15.0)	1881 (85.0)	*ref*.			
Yes	40 (18.6)	98 (81.4)	2.3 [1.6–3.4]	<0.001	2.6 [1.7–3.1]	<0.001
*Parent* ^c^						
Yes/No, I’m currently pregnant	273 (19.9)	1097 (80.1)	*ref*.			
No	100 (10.2)	882 (89.8)	0.5 [0.4–0.6]	<0.001	0.5 [0.4–0.7]	<0.001
*Adopted* ^c^						
No/I don’t know	358 (15.7)	1926 (84.3)	*ref*.			
Yes	13 (24.5)	40 (75.5)	1.7 [0.9–3.3]	0.09		
*Genetic condition in self*						
No	281 (13.5)	1804 (86.5)	*ref*.			
Yes	92 (34.5)	175 (65.5)	3.4 [2.5–4.5]	<0.001	2.9 [2.1–4.1]	<0.001
*Genetic condition in family*						
No	252 (13.6)	1601 (86.4)	*ref*.			
Yes	121 (24.2)	378 (75.8)	2.0 [1.6–2.6]	<0.001	1.3 [1.0–1.7]	0.09
*Self-reported health*						
Fair/poor/unknown	66 (16.2)	341 (83.8)	*ref*.			
Excellent/very good/good	307 (15.8)	1 638 (84.2)	1.0 [0.7–1.3]	0.8		

^a^Carrier tests were classified as PGT when:

(1) There was NO diagnosed genetic condition in themselves

(2) There was NO diagnosed genetic condition in their family

(3) When asked about why they had carrier testing, they indicated one or more of the following reasons:

a. I was planning to have children and wanted to know if there is a risk of passing on a genetic condition to my children

b. I wanted this information for my grandchildren

c. I thought I was at a higher risk because of my ethnic background

d. I was curious

e. I didn’t think about it, I just did it

^b^Age categories are structured to reflect the focus group categories from stage one of the Genioz research study

^c^These questions were optional in the survey

A second bivariate logistic regression was conducted to determine if carrier testing had an impact on the results. In this survey, 36 respondents identified they either had a personal or family history of a genetic condition and had indicated they had carrier testing only and for reproductive reasons; these records were excluded from analysis to see if they influenced relationships between variables and having had a PGT (*n* = 337). As shown in Supplementary Table 1, there appears to be no effect on the relationships when these respondents were removed from analysis.

The Pearson’s chi-squared test indicated there is a significant relationship between age and type of test for respondents who reported having undergone testing (*n* = 571). Results suggest respondents aged 20–49 are more likely to have had carrier testing (77.01, d*f* = 1, *P* < 0.0001) and respondents aged 50+ are more likely to have had ancestry testing (43.18, d*f* = 1, *P* < 0.0001).

### Perceived barriers to testing

**Fig. 2:**
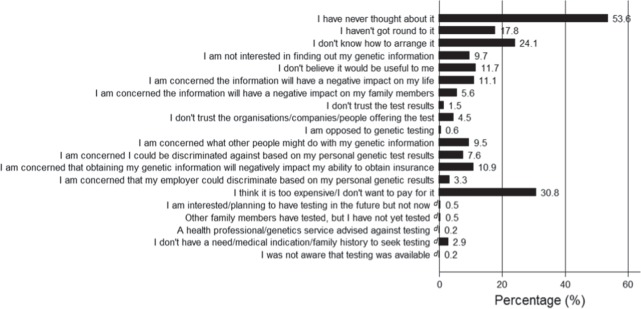
Reason/s reported by respondents for not having a genetic test (**a**–**c**) (*n* = 1918). **a** Wording from survey asks about “genetic” test. **b** Respondents could select more than one option. **c** Responses are in order they appeared in the survey. **d** Recoded categories from open-text responses

According to respondents who had not had testing at the time of completing the survey, more than 50% of respondents indicated that they had never thought about PGT. However, the largest barrier to pursuing testing was cost (Fig. 2). Additional barriers included: not knowing how to arrange such a test, potential negative impacts for self or family and concerns about third party access or discrimination.

### Seeking help with interpreting test results

**Fig. 3:**
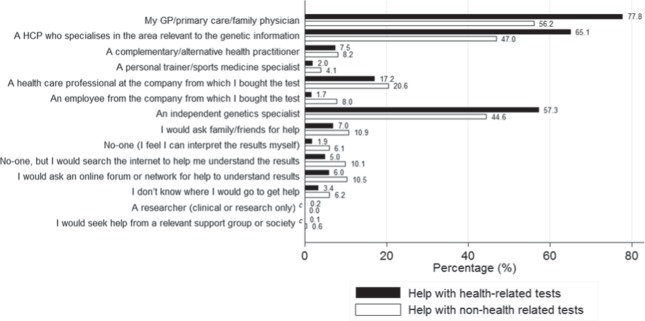
Respondents’ perception of who is appropriate to seek help from for test interpretation (**a**, **b**) (*n* = 2409). **a** Respondents could tick more than one response. **b** Options as they appeared in the survey. **c** New categories based on recoding open responses

Regardless of their prior experiences with testing, the top three sources people reported they would approach for help understanding their results (for both health and nonhealth tests) were: a GP/primary care/family physician; a health care professional who specializes in the relevant area; or an independent genetic specialist (for example, a clinical geneticist or a genetic counselor) (Fig. 3).

### Satisfaction with testing

At the end of the survey, respondents were given the opportunity to report their satisfaction with having had a genetic test. Out of the 529 respondents who reported having undergone testing and completed the survey, 396 (74.9%) were satisfied with their decision to undergo testing; 78 (14.7%) reported feeling neutral/unsure about their decision; 7 (1.3%) were not satisfied with their decision to undergo testing; and 48 (9.1%) reported that their satisfaction depended on the test they had if they had undergone more than one type of testing.

### Respondents changing their mind about genetic testing

**Fig. 4:**
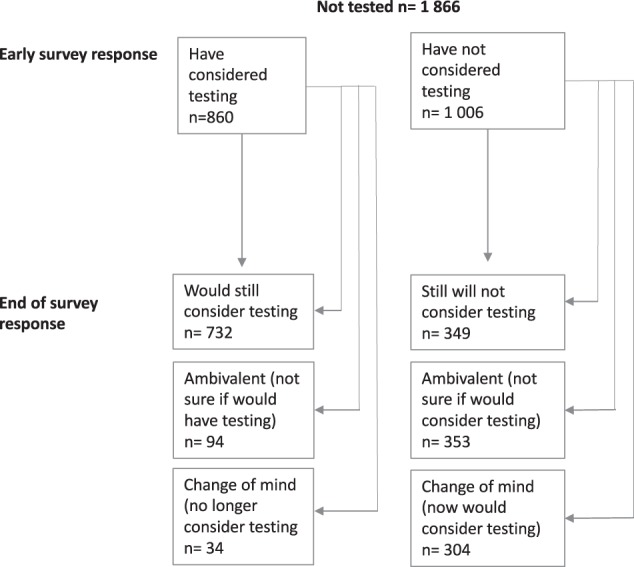
Respondents’ (not tested) end of survey responses to whether they would consider having a test in the future

At the end of the survey, respondents who had not previously had a genetic test were asked whether they would consider testing in the future and responses were compared to those given earlier in the survey. Of the 1866 who answered this question, 1081 (57.9%) did not change their mind about whether they would consider testing or not, and 785 (42.1%) did register a change in mind (see Fig. 4).

Of the 785 respondents who changed their mind, 657 respondents who initially *did not consider testing* changed their mind to either: now considering testing (*n* = 304, 46.3%); or to “unsure” if they would consider testing (*n* = 353, 53.7%). Respondents who changed their mind to now considering testing, indicated the types of tests they became interested in (could tick multiple options): for conditions that are serious but preventable (*n* = 252); carrier testing (*n* = 227); ancestry testing (*n* = 180); for conditions that are serious and not preventable (*n* = 171); pharmacogenetics (*n* = 170); nutrition/wellness (*n* = 158); for conditions that are not serious (*n* = 138); fitness testing (*n* = 136); testing for traits (*n* = 114); and relationship testing (*n* = 76).

The remaining 128 who changed their mind did so from initially saying *yes, they have considered* testing to either: no longer considering testing (*n* = 34, 26.6%) or “unsure” if they would consider testing (*n* = 94, 73.4%).

## Discussion

This Australian study aimed to collect data about knowledge, attitudes and experiences with PGT. Our sample is unique because it captures respondents with and without experiences of PGT when the visibility and availability of testing was just beginning to increase in Australia.

Statistical analysis of respondents’ experiences with PGT (excluding clinical testing) indicated an association with the following characteristics: being female, having a university education, and having a genetic condition diagnosed in oneself. These findings are similar to other quantitative research where: respondents were likely to have a family history of a genetic condition [21]; were highly educated; and from a high socioeconomic status [22]. However, it is important to note that question differences, along with recruitment strategies between cohorts prevent direct comparisons between the studies. Our findings offer a distinct Australian view, an international perspective that has been notably absent in systematic reviews of the empirical literature [23, 24].

The Genioz study offers a perspective on PGT that differs from the published literature for four reasons. First, Australia has a hybrid health care system, such that publicly funded health care is available to eligible individuals, but it can be supplemented by private health insurance [25]. Secondly, there is a federal prohibition on advertising prescription drugs and medical devices direct-to-consumers [26], which includes the provision of health-related direct-to-consumer genetic tests. In 2017, there was a change to Australian standards, such that for a laboratory to provide genetic susceptibility and genetic predisposition information for conditions, requires NATA (National Association of Testing Authorities; Australia) certification and registration to the Australian Register of Therapeutic Goods [27]. Thirdly, during the time the survey was available to respondents, an Australian-focused ancestry testing product was promoted via television, print and online advertising [2, 28]. This was in conjunction with a television program featuring Australian personalities exploring their ancestral origins with PGT [29]. This event coincided with the survey launch and subsequently, ancestry testing was the second most popular type of testing Genioz respondents had purchased. Finally, previous empirical research has found that Australians are more comfortable seeking health-related genetic information from a health care professional instead of a commercial company [9]. This may contribute to why health-related PGT was not reported as a popular type of testing by survey respondents.

While our sample recorded ancestry testing as the second most popular test type, the most reported by respondents was carrier testing. Findings indicate that in our sample, respondents aged 25–49 years were more likely to have pursued a form of carrier testing, while participants aged ≥50 years were most likely to have pursued ancestry testing. This potentially reflects goals and interests of the respective age groups; for example, family planning in the younger cohort and genealogical research in the older cohort. However, it should be noted that at present, there is not a recognized carrier screening program that forms a part of “regular” clinical care in Australia, but awareness of carrier testing and screening is increasing [30]. As a result, if a consumer wanted carrier testing in the absence of a family history, they are likely to seek a commercially offered and marketed test: a PGT.

A pivotal issue within this study was the difficulty of how to classify different types of genetic testing as a PGT or not PGT. Previous labeling of PGTs as either health- or nonhealth-related are no longer sufficient due in part to the increasing after-market applications that allow people to perform further analysis on their raw data [31]. Thus, boundaries are becoming blurred between health-related and nonhealth-related genomic information and the different ways it could be used [32]. These different uses of genomic information raise pragmatic concerns about who consumers can seek guidance and assistance from when they initially receive PGT results and/or additional results based on their own further analysis [3].

Issues can arise when consumers purchase genomic information from a commercial company and bring their results into a public health care system. For example, cost implications, misinterpretation of results, and limited expertize of nongenetic health professionals. Some companies do provide educational materials on their websites and details for their consumers to contact genetic counseling services (at their own expense). However, the resources can be variable. Within our sample, respondents reported that it was most appropriate to seek help with understanding PGT results from independent health professionals. A potential consequence of this approach means extra pressure on an already over-burdened health care system when PGTs are brought in by a consumer for further explanation/consultation [33]. The current support structures and systems will need to evolve to support these types of consultations. Extended findings from the Genioz study [34], along with scholarship in the UK and the US suggest genetic counselors are well placed to support consumers of PGT [35, 36].

While most research has focused on the experiences consumers have had with testing, there is still limited scholarship exploring views by consumers who have not had testing [37, 38]. From the change-of-mind data collected here, it appears the survey had an impact on a select group of respondents who reported a change in their contemplation about testing. It was beyond the scope of the study to measure whether the survey tool was an intervention, but it does suggest that the process of completing the survey increased awareness of PGT. Subsequent stages of the Genioz study [12] explore attitudes towards and experiences with PGT in greater depth, and how facilitated engagement may impact upon views on testing and the use of personal genomics.

Our survey findings are limited in several ways. Most respondents in this study were women. This is in part reflective of our online recruitment strategies, given that other studies have also reported that women were over-represented [38–40] and that they are more likely to complete surveys they have found through social media [41]. Also, possibly reflective of our recruitment strategy, 15% of our sample reported that at the time they completed the survey they were working in a genomics-focused role. The survey collected self-reported data, which has been shown to contain inaccuracies [42]. In addition, given the structure, order of content and the length of the survey, biases may also contribute to the discrepancies in the data. These data represent a cross-section of a single time point and may not be reflective of people’s changing values and perspectives. Finally, as Australia has a population distribution across a vast landscape, connectivity to the Internet in some regions is variable, despite a high reported use of the Internet according to recent census data [43]. As the survey was only offered online, this would have made it difficult for some areas to be accessed for sampling purposes.

In conclusion, this survey contributes novel findings to an international perspective on personal genomics. Aside from carrier testing, ancestry was the most popular type of PGT reported. As the demand for PGT in Australia increases, our findings are timely and relevant. Importantly, there appear to be blurring boundaries as to what constitutes a PGT and how the generated genomic information can be used. With this increasing PGT market in Australia there is a need for appropriate support and resources for consumers and health care professionals to help both understand and make sense of the results. The survey findings were used to contribute to the subsequent stages of the Genioz study and the development of engagement tools used in further research.

## Supplementary information

Supplementary Table 1

Supplementary Figure 1

Supplementary Figure 2

Supplementary Figure 3

Supplementary Figure 4

Supplementary Figure 5

Supplementary Figure 6
