# Supplementary material for: Australians’ views and experience of personal genomic testing: survey findings from the Genioz study
Source: Eur J Hum Genet. 2019 Jan 21;27(5):711–20. doi: 10.1038/s41431-018-0325-x (PMC6461785; doi:10.1038/s41431-018-0325-x)
Supplement: Supplementary file 1 — Supplementary Table 1 [file 41431_2018_325_MOESM1_ESM.docx]

Supplementary Table 1: Bivariate Logistic Regression and multivariate logistic regression analysis of demographics and testing experience with carrier testing excluded as a PGT *^a^*

| Variable | Had PGT  n (%) | Not had PGT  n (%) | Unadjusted odds ratio  [95% CI] | p-value | Adjusted odds ratio  [95% CI] | p-value |
| --- | --- | --- | --- | --- | --- | --- |
|  |  |  |  |  |  |  |
| Age *^b^* |  |  |  |  |  |  |
| 50+ | 216 (20.2) | 851 (79.8) | *ref.* |  |  |  |
| 25-49 | 113 (12.3) | 808 (87.7) | 0.10 [0.05-0.2] | <0.001 |  |  |
| 18-24 | 8 (2.4) | 320 (97.6) | 0.2 [0.09-0.4] | <0.01 |  |  |
|  |  |  |  |  |  |  |
| Age |  |  |  |  |  |  |
| 18-49 | 121 (9.7) | 1 128 (90.3) | *ref.* |  |  |  |
| 50+ | 216 (20.2) | 851 (79.8) | 2.4 [1.9-3.0] | <0.001 | 2.7 [2.0-3.6] | <0.001 |
|  |  |  |  |  |  |  |
| Sex |  |  |  |  |  |  |
| Male | 81 (11.7) | 610 (88.3) | *ref.* |  |  |  |
| Female | 255 (15.8) | 1357 (84.2) | 1.4 [1.1-1.8] | 0.01 | 1.5 [1.1-2.0] | 0.006 |
|  |  |  |  |  |  |  |
| SEIFA (IRSAD) |  |  |  |  |  |  |
| 1^st^ to 4^th^ quintiles | 194 (13.6) | 1 235 (86.4) | *ref.* |  |  |  |
| 5^th^ quintile | 144 (16.4) | 730 (85.4) | 1.2 [0.99-1.6] | 0.07 |  |  |
|  |  |  |  |  |  |  |
| Education |  |  |  |  |  |  |
| Never studied at university | 109 (11.2) | 863 (88.8) | *ref.* |  |  |  |
| Currently studying/completed university | 226 (16.9) | 1 115 (83.1) | 1.6 [1.3-2.1] | <0.001 | 2.4 [1.8-3.1] | <0.001 |
|  |  |  |  |  |  |  |
| Working in genomics and/or life sciences |  |  |  |  |  |  |
| No | 303 (13.9) | 1 881 (86.1) | *ref.* |  |  |  |
| Yes | 34 (25.8) | 98 (74.2) | 2.2 [1.4-3.2] | <0.001 | 2.6 [1.7-4.1] | <0.001 |
|  |  |  |  |  |  |  |
| Parent *^c^* |  |  |  |  |  |  |
| Yes/No, I’m currently pregnant | 249 (18.5) | 1 097 (81.5) | *ref.* |  |  |  |
| No | 88 (9.1) | 882 (90.9) | 0.4 [0.3-0.6] | <0.001 | 0.5 [0.4-0.7] | <0.001 |
|  |  |  |  |  |  |  |
| Adopted *^c^* |  |  |  |  |  |  |
| No/I don’t know | 323 (14.4) | 1 926 (85.6) | *ref.* |  |  |  |
| Yes | 13 (24.5) | 40 (75.5) | 1.9 [1.0-3.7] | 0.04 |  |  |
|  |  |  |  |  |  |  |
| Genetic condition in self |  |  |  |  |  |  |
| No | 251 (12.2) | 1 804 (87.8) | *ref.* |  |  |  |
| Yes | 86 (33.0) | 175 (67.0) | 3.5 [2.6-4.7] | <0.001 | 3.0 [2.1-4.2] | <0.001 |
|  |  |  |  |  |  |  |
| Genetic condition in family |  |  |  |  |  |  |
| No | 228 (12.5) | 1 601 (87.5) | *ref.* |  |  |  |
| Yes | 109 (22.4) | 378 (77.6) | 2.0 [1.6-2.6] | <0.001 | 1.3 [0.9-0.8] | 0.12 |
|  |  |  |  |  |  |  |
| Self-reported health |  |  |  |  |  |  |
| Fair/poor/unknown | 65 (16.0) | 341 (84.0) | *ref.* |  |  |  |
| Excellent/very good/good | 272 (14.2) | 1 638 (85.8) | 1.1 [0.9-1.5] | 0.4 |  |  |

*a –* Carrier tests were NOT classified as a PGT when respondents had indicated the following:

1. The respondent indicated they either had a diagnosed genetic condition themselves and/or there was a diagnosed genetic condition in their family
2. The respondent indicated they had a genetic test and identified they had carrier testing because of a family history
3. When respondents had carrier testing through a research program.

*b –* Age categories are structured to reflect the focus group categories from stage one of the Genioz research study

*c* – These questions were optional in the survey
