# Supplementary material for: Australians’ views and experience of personal genomic testing: survey findings from the Genioz study
Source: Eur J Hum Genet. 2019 Jan 21;27(5):711–20. doi: 10.1038/s41431-018-0325-x (PMC6461785; doi:10.1038/s41431-018-0325-x)
Supplement: Supplementary file 2 — Supplementary Figure 1 [file 41431_2018_325_MOESM2_ESM.pdf]

Supplementary Figure 1: Cross-sectional survey sections[a]

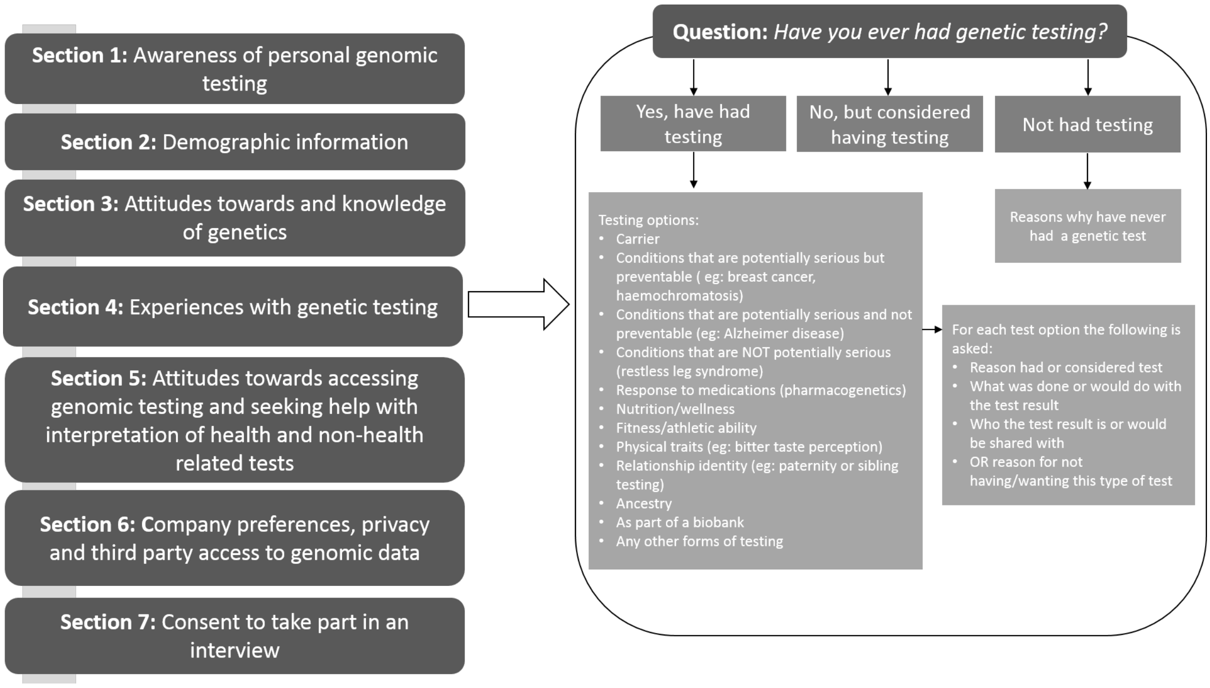

[a]Full survey is available on request
