# Supplementary figures and images for: Australians’ views and experience of personal genomic testing: survey findings from the Genioz study
Source: Eur J Hum Genet. 2019 Jan 21;27(5):711–20. doi: 10.1038/s41431-018-0325-x (PMC6461785; doi:10.1038/s41431-018-0325-x)

Supplementary Figure 2: Survey advertising and recruitment strategy

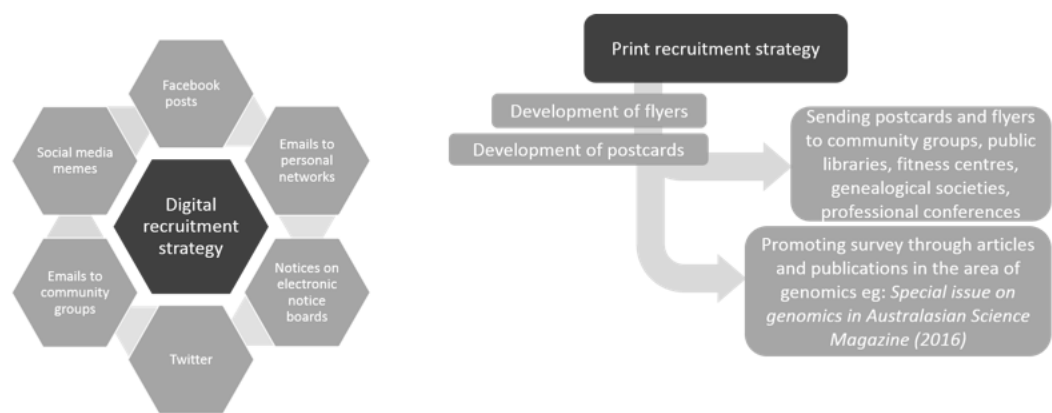

Supplement: Supplementary file 3 — Supplementary Figure 2 [file 41431_2018_325_MOESM3_ESM.pdf]
