# Supplementary material for: Australians’ views and experience of personal genomic testing: survey findings from the Genioz study
Source: Eur J Hum Genet. 2019 Jan 21;27(5):711–20. doi: 10.1038/s41431-018-0325-x (PMC6461785; doi:10.1038/s41431-018-0325-x)
Supplement: Supplementary file 4 — Supplementary Figure 3 [file 41431_2018_325_MOESM4_ESM.pdf]

Supplementary Figure 3. SEIFA[a] Comparisons between Genioz data (n=2 797) and Australian Bureau of Statistics 2011 Data

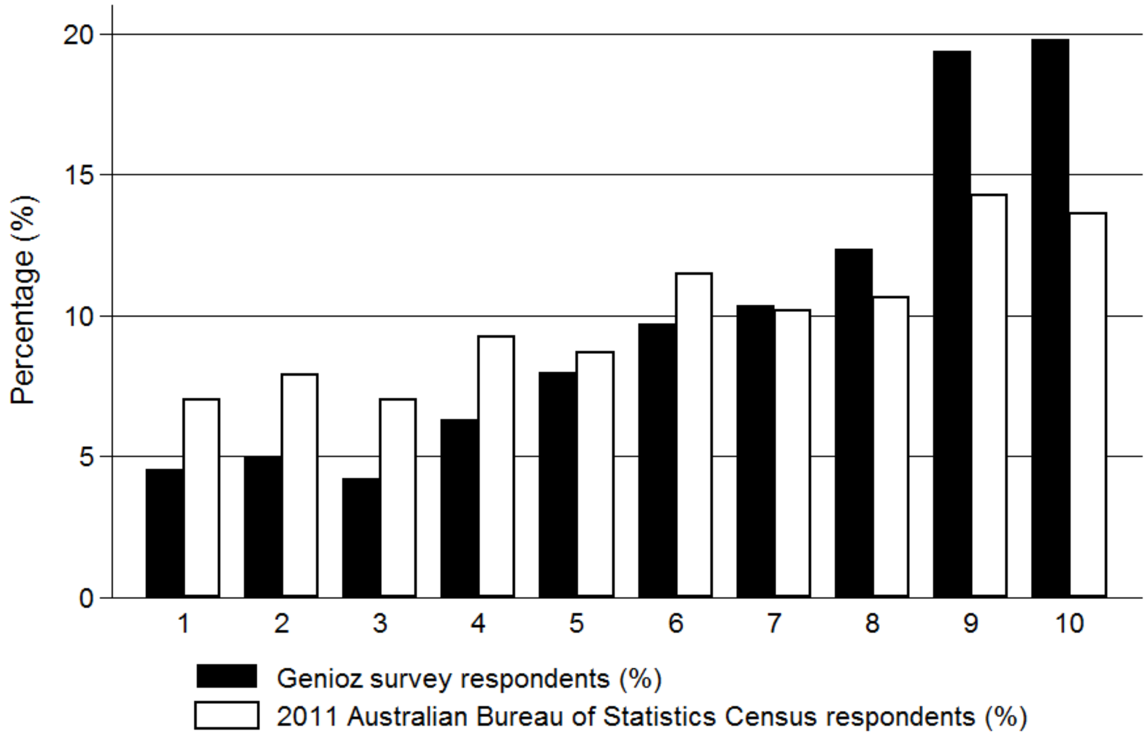

[a] SEIFA (IRSAD) – Socio-Economic Indexes for Areas (Index of Relative Socio-Economic Advantage and Disadvantage) – ranks areas in Australia according to relative socio-economic advantage and disadvantage. These indices are based on information from the five-yearly census. For brevity, the deciles as displayed above from the ABS have been collapsed to quintiles, as reported in Table 1.
