# Supplementary material for: Australians’ views and experience of personal genomic testing: survey findings from the Genioz study
Source: Eur J Hum Genet. 2019 Jan 21;27(5):711–20. doi: 10.1038/s41431-018-0325-x (PMC6461785; doi:10.1038/s41431-018-0325-x)

Supplementary Figure 4: Age of Genioz respondents (n=2 819) compared with Australian Bureau of Statistics 2011 census data

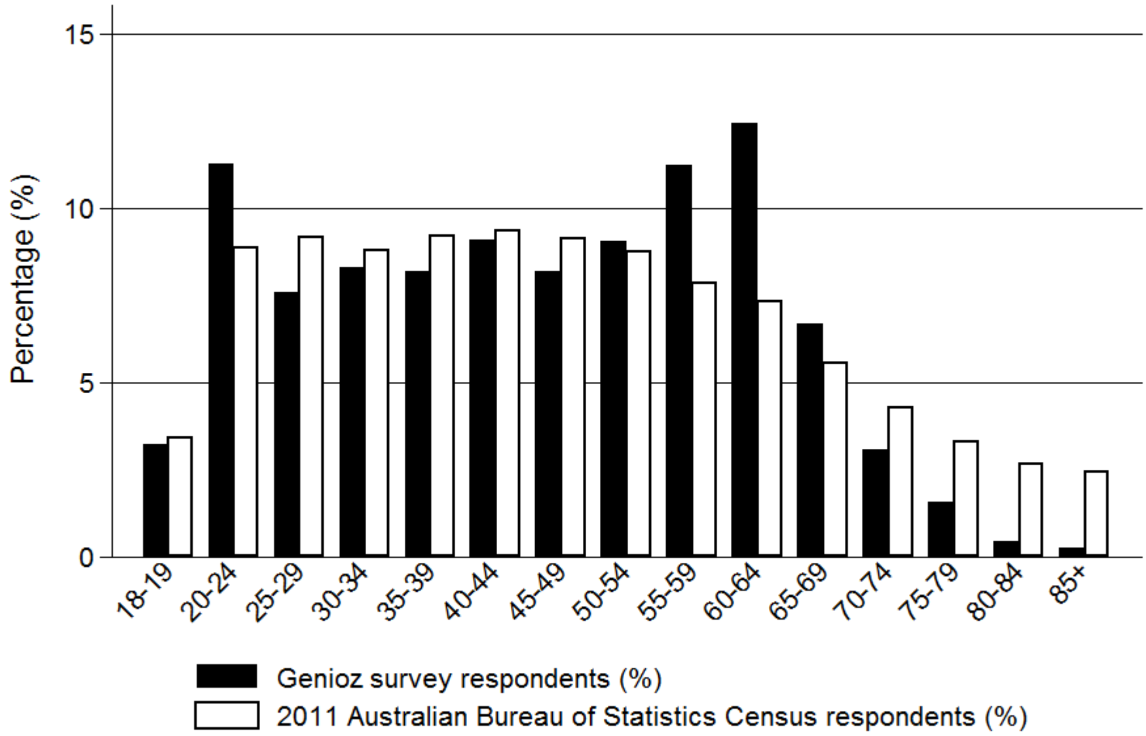

Supplement: Supplementary file 5 — Supplementary Figure 4 [file 41431_2018_325_MOESM5_ESM.pdf]
