# Supplementary material for: Australians’ views and experience of personal genomic testing: survey findings from the Genioz study
Source: Eur J Hum Genet. 2019 Jan 21;27(5):711–20. doi: 10.1038/s41431-018-0325-x (PMC6461785; doi:10.1038/s41431-018-0325-x)

Supplementary Figure 5. Boxplot of the average median score out of 15 for the knowledge questions for all respondents (n=2 633); including a breakdown of those who have had testing (n=571) and those without testing (n=2 062)

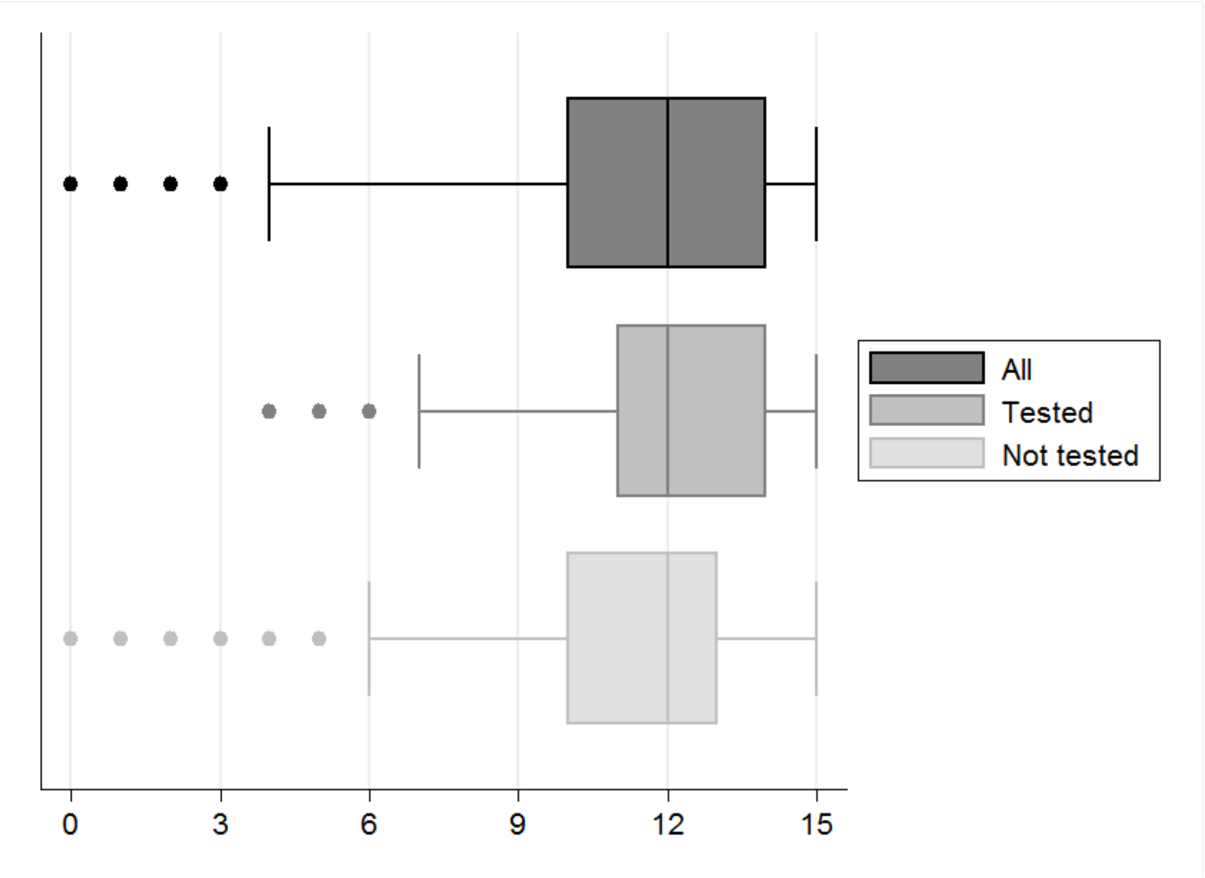

Supplement: Supplementary file 6 — Supplementary Figure 5 [file 41431_2018_325_MOESM6_ESM.pdf]
