# Supplementary material for: Australians’ views and experience of personal genomic testing: survey findings from the Genioz study
Source: Eur J Hum Genet. 2019 Jan 21;27(5):711–20. doi: 10.1038/s41431-018-0325-x (PMC6461785; doi:10.1038/s41431-018-0325-x)
Supplement: Supplementary file 7 — Supplementary Figure 6 [file 41431_2018_325_MOESM7_ESM.pdf]

Supplementary Figure 6: Reported ways testing was arranged (n=571)[a,b]

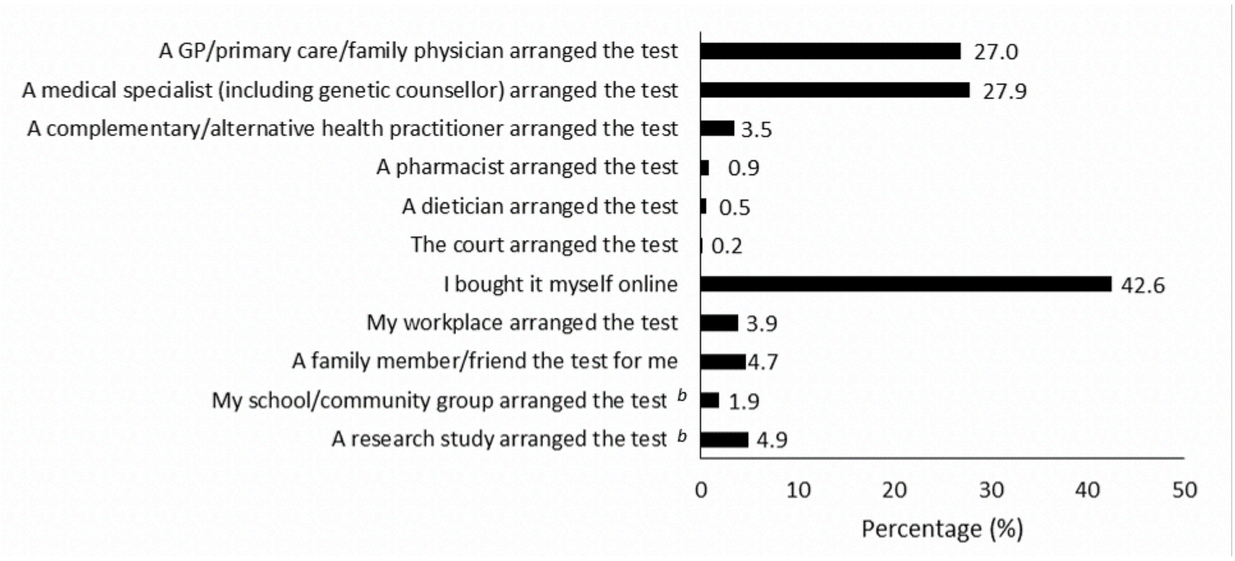

[a] – While there was n=571 completed records for this question, respondents with experience of more than one test type could select the different ways in which they accessed testing

[b] – The order of options are as they appeared in the survey. These test types are new categories developed during analysis, based on open-ended responses
